# Assessing Cardiometabolic Health Risk Among U.S. Children Who Live in Grandparent-Headed Households

**DOI:** 10.1093/geroni/igaa057.1689

**Published:** 2020-12-16

**Authors:** MinKyoung Song, Karen Lyons, Laura Hayman, Nathan Dieckmann, Carol Musil

**Affiliations:** 1 Oregon Health & Science University, Portland, Oregon, United States; 2 Boston College, Chestnut Hill, Massachusetts, United States; 3 University of Massachusetts Boston, Boston, Massachusetts, United States; 4 Case Western Reserve University, Cleveland, Ohio, United States

## Abstract

Many interventions have been designed to leverage parent-caregivers as change agents for improving children’s cardiometabolic health (CMH), however very few have been designed to leverage grandparent-caregivers for that purpose. This is surprising since there has been a steady increase in children living in grandparent-headed households. As a first step in assessing the potential impact of interventions with grandparent-caregivers, we used data from the National Survey of Children’s Health (2018) to compare CMH measures in children living in grandparent-headed households with CMH measures in children living in parent-headed households. Our hypothesis was that CMH risk might be higher in grandparent households – given that research shows that grandparents taking over caregiving from parents is associated with worse overall health outcomes for both grandparents and their grandchildren. Additionally, since research indicates that children who experience ≥ 4 adverse childhood experiences (ACEs) have significantly worse health outcomes, we assessed levels of ACEs. Our analytic sample included children aged 10-17 years (n=14,941). Adjusting for age, sex, race/ethnicity, and health insurance coverage status, children living in grandparent households were more likely to be obese (Adjusted Odds Ratio [95% confidence interval]= 2.04 [1.02, 4.09]), exposed to secondhand smoke (2.32 [1.49, 3.59]), and less likely to meet recommended age-appropriate standards for sleep (0.42 [0.27, 0.67]). The children living in grandparent households were more likely to experience ≥ 4 ACEs (8.59 [5.42, 13.62]). Our results provide indirect evidence that interventions with grandparent-caregivers may be particularly critical for improving CMH risk in families.

